# Modelling and mapping the intra-urban spatial distribution of *Plasmodium falciparum* parasite rate using very-high-resolution satellite derived indicators

**DOI:** 10.1186/s12942-020-00232-2

**Published:** 2020-09-21

**Authors:** Stefanos Georganos, Oscar Brousse, Sébastien Dujardin, Catherine Linard, Daniel Casey, Marco Milliones, Benoit Parmentier, Nicole P. M. van Lipzig, Matthias Demuzere, Tais Grippa, Sabine Vanhuysse, Nicholus Mboga, Verónica Andreo, Robert W. Snow, Moritz Lennert

**Affiliations:** 1 Department of Geoscience, Environment & Society, Université Libre de Bruxelles, 1050 Brussels, Belgium; 2 Department of Earth and Environmental Sciences, KU Leuven, Celestijnenlaan 200E, 3001 Louvain, Belgium; 3 Institute of Life, Earth and Environment, University of Namur, 5000 Namur, Belgium; 4 Department of Geography, University of Namur, Rue de Bruxelles 61, 5000 Namur, Belgium; 5 Senator George J. Mitchell Center for Sustainability Solutions, University of Maine, 5710 Norman Smith Hall, Orono, ME 04469-5710 USA; 6 Department of Geography, University of Mary Washington, 1301 College Avenue, Fredericksburg, VA 22401 USA; 7 Department of Geography, Ruhr-University Bochum, Bochum, Germany; 8 Instituto de Altos Estudios Espaciales “Mario Gulich”. Comisión Nacional de Actividades Espaciales (CONAE), Universidad Nacional de Córdoba (UNC), Córdoba, Argentina; 9 Consejo Nacional de Investigaciones Científicas y Técnicas (CONICET), Buenos Aires, Argentina; 10 Population and Health Unit, Kenya Medical Research Institute/Wellcome Trust Research Programme, Nairobi, Kenya; 11 Centre for Tropical Medicine and Global Health, Nuffield Department of Clinical Medicine, University of Oxford, Oxford, UK

**Keywords:** Urban malaria, Random forest, Kampala, Dar es Salaam, Remote sensing, Population

## Abstract

**Background:**

The rapid and often uncontrolled rural–urban migration in Sub-Saharan Africa is transforming urban landscapes expected to provide shelter for more than 50% of Africa’s population by 2030. Consequently, the burden of malaria is increasingly affecting the urban population, while socio-economic inequalities within the urban settings are intensified. Few studies, relying mostly on moderate to high resolution datasets and standard predictive variables such as building and vegetation density, have tackled the topic of modeling intra-urban malaria at the city extent. In this research, we investigate the contribution of very-high-resolution satellite-derived land-use, land-cover and population information for modeling the spatial distribution of urban malaria prevalence across large spatial extents. As case studies, we apply our methods to two Sub-Saharan African cities, Kampala and Dar es Salaam.

**Methods:**

Openly accessible land-cover, land-use, population and OpenStreetMap data were employed to spatially model *Plasmodium falciparum* parasite rate standardized to the age group 2–10 years (PfPR_2–10_) in the two cities through the use of a Random Forest (RF) regressor. The RF models integrated physical and socio-economic information to predict PfPR_2–10_ across the urban landscape. Intra-urban population distribution maps were used to adjust the estimates according to the underlying population.

**Results:**

The results suggest that the spatial distribution of PfPR_2–10_ in both cities is diverse and highly variable across the urban fabric. Dense informal settlements exhibit a positive relationship with PfPR_2–10_ and hotspots of malaria prevalence were found near suitable vector breeding sites such as wetlands, marshes and riparian vegetation. In both cities, there is a clear separation of higher risk in informal settlements and lower risk in the more affluent neighborhoods. Additionally, areas associated with urban agriculture exhibit higher malaria prevalence values.

**Conclusions:**

The outcome of this research highlights that populations living in informal settlements show higher malaria prevalence compared to those in planned residential neighborhoods. This is due to (i) increased human exposure to vectors, (ii) increased vector density and (iii) a reduced capacity to cope with malaria burden. Since informal settlements are rapidly expanding every year and often house large parts of the urban population, this emphasizes the need for systematic and consistent malaria surveys in such areas. Finally, this study demonstrates the importance of remote sensing as an epidemiological tool for mapping urban malaria variations at large spatial extents, and for promoting evidence-based policy making and control efforts.

## Introduction

Unprecedented rates of rural–urban migration and natural population increase in sub-Saharan Africa (SSA) have dramatically affected urban environments [1]. Low income housing has not kept up with population growth which has contributed to widely varying physical and socio-economic landscapes within cities where formal and informal settlements coexist [2]. Informal settlements are often characterized by residential areas where land tenure is not recognized by authorities, housing quality is sub-standard and access to several basic services is lacking [3–5]. These rapidly transforming environments, have an impact upon urban health, such as the risk of infection with vector-borne diseases [6, 7].

While malaria has widely been known as a rural disease, uncontrolled urbanisation has altered urban landscapes in ways that may increasingly support vector breeding, making the disease to be persistent in urban settings [7–9]. One reason for this is the increased likelihood of breeding sites for mosquitoes of the genus *Anopheles*, the vectors of the *Plasmodium falciparum* parasite [10]. Previous work has highlighted the focal nature of urban malaria and its link with human activities. For example, the development of urban agricultural areas, irrigation schemes, market gardens, open water storage, or even open excavation during the construction of building sites and roads have led to rain-fed breeding sites associated with increased malaria prevalence [7–9, 11–16]. Furthermore, the functional organization of cities can influence the heterogeneity of urban malaria risk. Areas with peripheral housing settlements and a central business district may exhibit different malaria patterns compared to those with business districts located on the periphery and housing located centrally.

The social vulnerability of a population, which can vary spatially, is also expected to affect the ability of a population to cope with the burden of malaria [17]. Previous work has shown that malaria prevalence can be significantly higher in informal settlements than in other urban landscapes due to poor housing infrastructure, lack of bed nets, and inadequate financial resources to buy anti-malarial drugs, among others [18, 19]. Hence, given the same levels of vector density, two communities with significantly different levels of income and education might have significantly different prevalence levels of malaria. These variations, often dominated by differences between planned and unplanned settlements, might explain the clustered nature of urban malaria in SSA cities [20]. Indeed, as noted by Taubenböck [21], the physical urban surface reflects the underlying social processes that developed it. It would be reasonable to assume that spatial malaria models capturing a combination of the physical surface and the population’s socio-economic levels are more informative than those only relying on a purely physical representation of the land cover.

Satellite images can contribute a vast amount of information for modeling and mapping malaria prevalence at the city scale. The intra-urban component of malaria dynamics, however, has neither been part of continental malaria risk mapping initiatives nor considered part of most national control strategies [22–25]. Satellite imagery can provide valuable input for epidemiological models such as detailed land-cover (LC), land-use (LU) maps, as well as socioeconomic indicators and population distribution maps. Recent research has demonstrated that several moderate or high-resolution geospatial and/or satellite-derived features could distinguish areas of higher malaria risk within the urban settings of Dar es Salaam, Tanzania [6, 11], Ouagadougou, Burkina Faso [26] and Dakar, Senegal [9], often due to the differences in building density and vegetation type coverage.

In this paper, we model and map the spatial distribution of malaria prevalence across two SSA cities—Dar es Salaam and Kampala, using very-high-resolution (VHR) satellite indicators and machine learning techniques. We investigate the use of VHR LC and LU classes as a composite of both physical and socio-economic information to predict malaria prevalence. We then examine the use of human population products to adjust our estimates according to the underlying population. Our research objectives can be summarized as follows:

1. Assessing the potential of VHR satellite-derived LC, LU and population information for modeling urban malaria prevalence.
2. Exploring their utility as tools to map and highlight intra-urban variations of malaria prevalence across large extents.

## Methods

### Case studies

#### Dar es Salaam, Tanzania

Dar Es Salaam is the former capital of Tanzania with an estimated population exceeding five million, and one of the fastest growing cities in the world [27]. According to recent estimates, 75% of the residential population lives in informal settlements where only a small part of the urban fabric is planned [28–30]. Malaria is endemic in the city, with over one million cases reported annually [6, 11, 31]. The Urban Malaria Control Project (UMCP) has been responsible for a large part of the efforts to control malaria transmission in the city through ground-based sampling and monitoring [6, 14, 19, 32, 33]. While entomological inoculation rates in urban areas can in general be considered lower than rural regions, this might not hold true in urban slums, as these are usually built around environments that favor mosquito vector breeding [19]. The dominant vector species in Dar es Salaam, come from the *Anopheles gambiae* complex with a smaller contribution coming from *Anopheles funestus* [19, 34]. *Anopheles gambiae* are usually found in small bodies of freshwater while *Anopheles funestus* is frequently encountered in permanent water bodies such as wetlands and marshes [19]. Additionally, *Anopheles arabiensis* of the gambiae complex are increasingly feeding outdoors, an adaptation to the high levels of bed nets usage and house protection [32, 35]. Analyzing samples from health care facilities in and around Dar es Salaam, Wang et al. [36] found that the differences of malaria prevalence between the urban–rural spectrum in the city were low. In addition, Kabaria et al. [6] identified increased risk across riparian vegetation and wetlands in the city.

#### Kampala, Uganda

Kampala is the capital and main economic center of Uganda, with a population of over 1.5 million [37]. Similar to Dar es Salaam, Kampala is growing rapidly every year at a rate of about 5% [38]. Informal settlements house roughly 60% of the urban residents [39, 40]. Malaria is endemic in the region, and previous research has noted significant spatial variations within the city associated with the residential characteristics of sampled locations such as the water sources utilized by a household [41]. The majority of the malaria vectors in Kampala are of the *Anopheles gambiae* complex, with a smaller population of *Anopheles funestus* vectors [42]. Informal settlements have been built in vicinity of marshes, streams and swamps because of their high viability for urban agriculture [43]. This influences the intra-urban spatial heterogeneity and is consistently implicated in increased malaria prevalence in these areas [44, 45]. In addition, the sanitation conditions of the city’s slums tend to deteriorate during the peak of the wet season in which malaria transmission intensifies [46]. In a study by Mukasa [18], it was shown that about 45% of the interviewed mothers from the Bwaese slum in Kampala, were not in possession of a bed net indicating high inability to cope with the burden of malaria.

### Satellite derived indicators

#### Land-cover (LC)

**Fig. 1:**
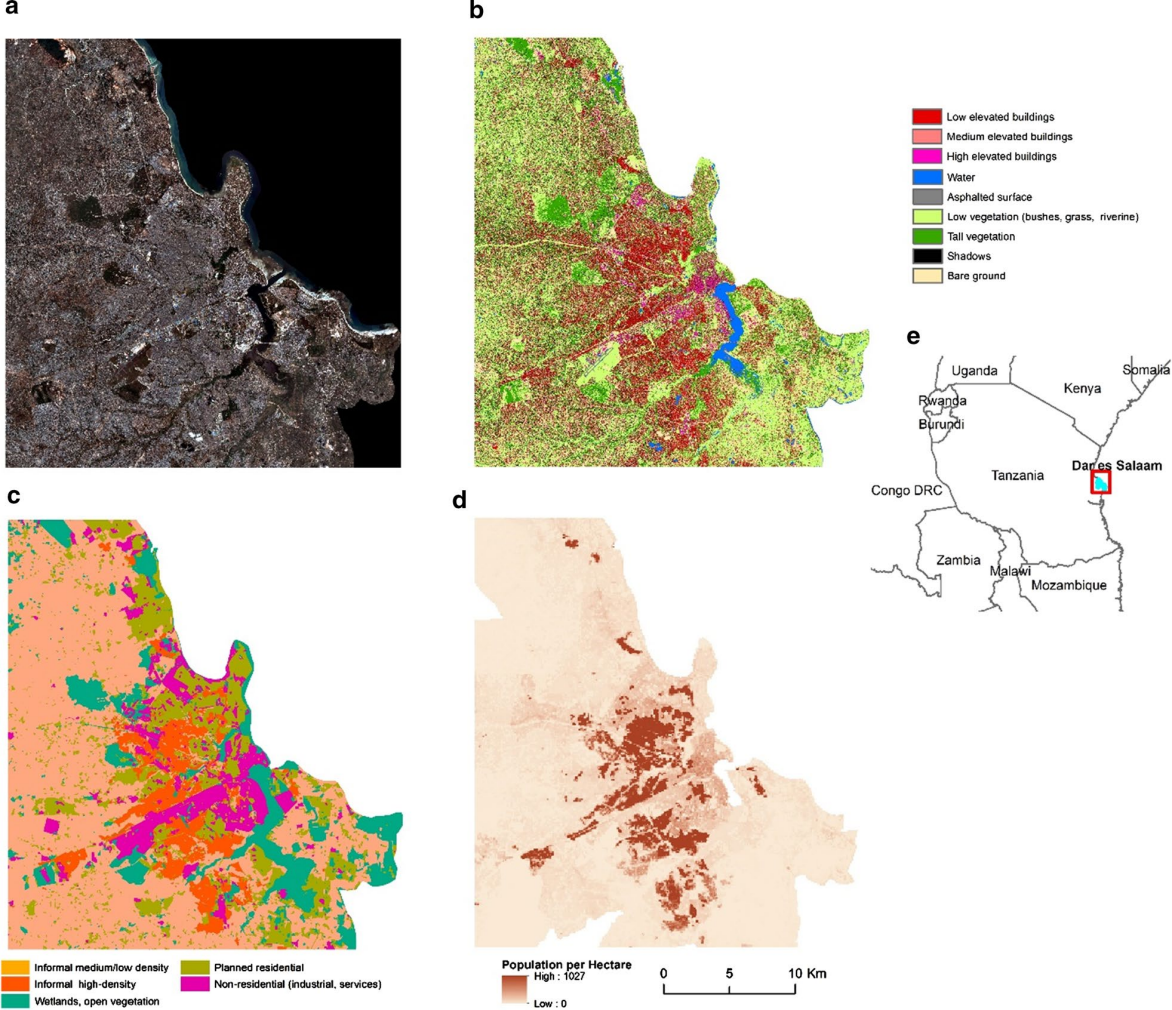
**a** Pleiades satellite imagery of Dar es Salaam—RGB natural color composite, **b** land cover (0.5 m resolution), **c** land use at the street block level, **d** population counts per hectare and **e** location map of Dar es Salaam within Tanzania

**Fig. 2:**
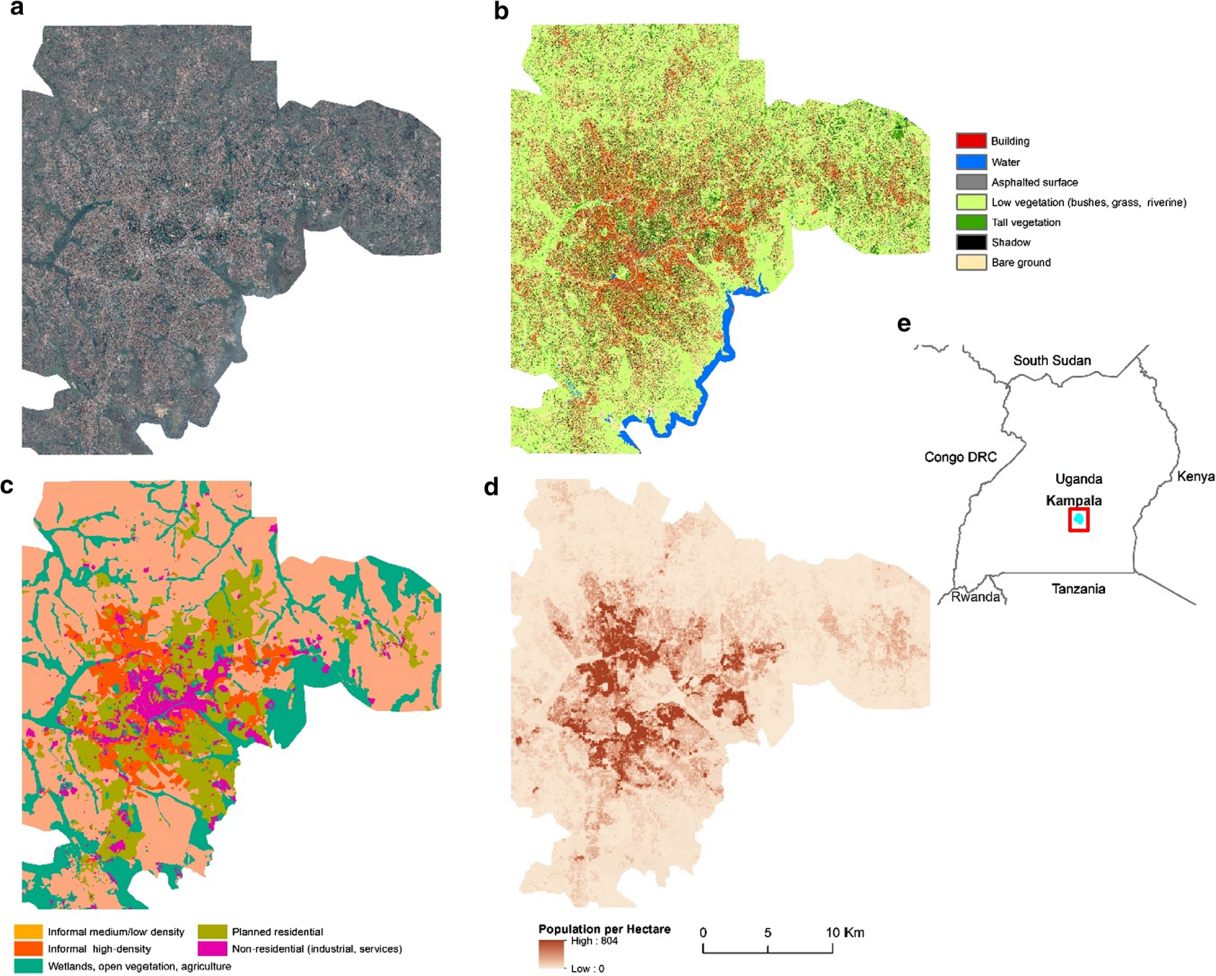
**a** Pleiades satellite imagery of Kampala—RGB natural color composite, **b** land cover (0.5 m resolution), **c** land use at the street block level, **d** population counts per hectare and **e** location map of Kampala within Uganda

The LC maps (50 cm resolution) used in this study were produced through a combination of Computer Assisted Photo Interpretation, Geographic Object Based Image Analysis GEOBIA and machine learning algorithms through open-access software ([47–49]; Additional file 1), and are openly accessible through the Zenodo scientific repository [50, 51]. The classifications were based on Pleiades satellite imagery of Kampala (collected in February 2013) and Dar es Salaam (stereo-images collected in March and January 2016 and July 2018). The LC of Kampala exhibited an overall accuracy of 86% (7 classes), while that of Dar es Salaam, an overall accuracy of 90% (9 classes). The accuracy metrics were the result of an assessment through out of bag error of a random forest (RF) classifier trained and validated at the date of acquisition. The building class in Dar es Salaam LC was further subdivided into three height subclasses due to the availability of photogrammetrically generated height elevation data [52]. The complete LC legend is shown in Figs. 1 and 2.

#### Land-use (LU)

The LU maps utilized here were the outcome of a processing chain involving the computation of LC-based spatial metrics derived from the maps mentioned above and information derived from OpenStreetMaps (OSM) [53]. Linear elements extracted from OSM such as the street network and various parcel types were imported into a PostGIS database and processed to create street-block polygons. Subsequently, a machine learning classifier assigned a LU value to each street-block through supervised training. The complete processing chain was a reproduction of the work by Grippa et al. ([54]; Additional file 1). The LU classification allowed for the classification of the urban surface according to its different urban functions (i.e., residential/non-residential). The residential classes were categorized into either formal or informal settlements. The complete LU legend is shown in Figs. 1 and 2.

#### Population density

We used high-resolution population maps (100-m resolution) that were constructed through population disaggregation algorithms trained on census data, and using the LC and LU data as input [55, 56] and Additional file 1). The validation of the population models demonstrated R^2^ values of 0.63 and 0.77 for Dar es Salaam and Kampala, respectively, which is in line with state-of-the-art results of similar studies [55–57].

#### Ancillary data

To complement the previous datasets, we extracted the Normalized Difference Vegetation Index (NDVI) from the raw VHR satellite images, and terrain height information (30 m resolution) from the NASA/NGA Shuttle Radar Topography Mission (SRTM) [58]. In the case of Dar es Salaam, OSM vector features such as wetlands, streams and rivers were used due to the high level of detailed information available for the city, in a large part thanks to community mapping projects. For instance, ‘Ramani Huria’, one of the largest community projects in Dar es Salaam aiming to mitigate hazard and flooding risk, has mapped detailed urban infrastructure such as the drainage network and buildings for more than 4 million people in the city since 2018 [29]. Table 1 summarizes the complete set of variables examined.

**Table 1:** Predictive variables investigated in each city

	Kampala	Dar es Salaam	Type
Land cover			
Low vegetation (humid, riparian, grasses, bushes)	X	X	Proportion
Tall vegetation	X	X	Proportion
Bare ground (or dried out vegetation)	X	X	Proportion
Water	X	X	Proportion
Building	X		Proportion
Low elevated building		X	Proportion
Medium elevated building		X	Proportion
High elevated building		X	Proportion
Land use			
Planned residential	X	X	Proportion
Informal residential − high density	X	X	Proportion
Informal residential − medium/low density	X	X	Proportion
Wetlands, streams, marshes, rivers (mixed class)	X		Distance
OSM			
Wetlands		X	Distance
Rivers		X	Distance
Streams		X	Distance
Ancillary features			
Population per hectare	X	X	Average
Normalized difference vegetation index	X	X	Average
Terrain elevation	X	X	Average

### *Plasmodium falciparum* prevalence data

**Fig. 3:**
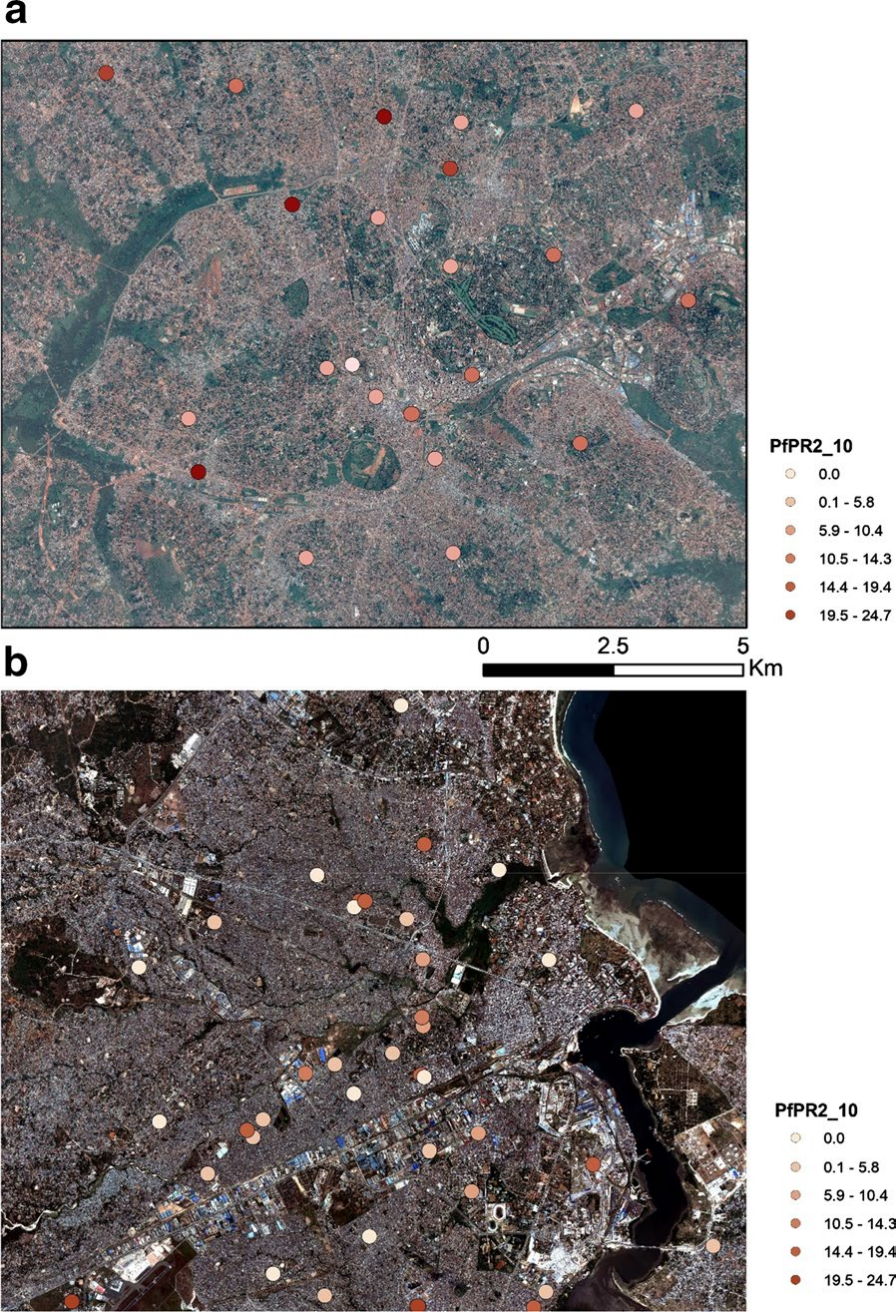
Overview of geolocated malaria surveys over a subset of the urban extent of **a** Kampala and **b** Dar es Salaam

Data of community surveys were extracted from an open access online database [59] that accompanied the publication of changing malaria prevalence across sub-Saharan Africa since 1900 [60]. From the available pool of surveys, we included those that have high degrees of spatial accuracy of the survey location (GPS coordinates or Google Earth validation) and consistent metadata information. Because different surveys often cover different age ranges, each parasite rate was standardized to the age group 2–10 (PfPR_2_10_) [6, 61, 62]. As mentioned by Smith et al. [63], PfPR_2_10_ combines reliable epidemiological and statistical properties beneficial for multi-survey comparison and analysis. The temporal range of selected samples was 2005-2014, resulting in 39 surveys (at 38 unique locations) for Kampala and 90 surveys (at 57 unique locations) for Dar es Salaam (Fig. 3). In Dar es Salaam, 27 (30%) school surveys were included, undertaken in 2014. In Kampala, 21 school surveys (54%) were included, with 20 of them undertaken in 2014. The average survey sample size in Kampala and Dar es Salaam, is 82 and 175, among individuals aged 0–16 years old, respectively. All surveys were random selections of communities or schools. Further information regarding the key characteristics of the malaria dataset can be found in Additional file 2. The mean PfPR_2_10_ values were 6.76% and 7.76% for Kampala and Dar es Salaam, respectively. 17.6% of the data points reported zero PfPR_2_10_. Finally, we used 1-kilometer buffers around each geolocated survey to extract aggregated values for each predictor mentioned previously in Table 1 (i.e. proportions for categorical features, mean values for continuous ones and the mean distance to the “Wetland”, “River” and “Stream” classes), similar to research employing survey data and geographical variables [6, 64]. Even though there was a temporal mismatch between the satellite imagery and the malaria data, we presume a degree of stationarity across the main urban extent as most of the LU changes in SSA cities are characterized mostly by expansion rather than transition, and that the malaria data are likely to be representative of land use/ecology in the period of the satellite imagery, as done in similar studies [6]. Moreover, the collapse of the temporal dimension was inevitable due to the limited sample size and was the only means to increase spatial coverage.

### Modelling and quality assessment methods

To train the *PfPR*_*2_10*_ models, we used a random forest (RF) regressor which has shown to be resilient to overfitting, capturing non-linear relationships and appropriate for heavily contextual models [65]. RF is an ensemble of regression decision trees, trained on random data bootstraps (bagging). In a standard RF configuration, each computed tree is trained using a random sub-sample of about 70% of the initial data. The average prediction of all computed trees is used as the final output [65]. The hyper parameters that require fine-tuning in RF are (i) the number of considered features for each decision split in each tree (feature bagging) and (ii) the total number of decision trees built. In this study, the former was determined through cross validation (value of 1), while the latter was set at a computationally efficient number (1000) through the R software’s caret package [66]. To create the final predictive models, we employed feature selection methods, namely the Variable Selection with Random Forests (VSURF) algorithm [67]. VSURF is a well-documented and robust variable selection procedure that uses iterative and nested RF models to identify variables contributing to the task at hand eliminating useless, noisy and/or redundant features. By creating more parsimonious models, the model performance may increase as several studies have shown [68, 69].

For the evaluation metrics, we reported the Root Mean Squared Error (RMSE), Mean Absolute Error (MAE) and the Predicted Coefficient of Determination (Predicted R^2^). Given the relatively small sample sizes, and to reduce the prediction bias, we made use of a bootstrap approach [70]. The train and test data were split in an 80:20 ratio using stratified random sampling through 100 simulations and reported on the average RMSE, MAE and R^2^ values. The sampling was stratified by the survey type (i.e., samples from a particular study) to make sure that the training and testing data distributions were similar. Finally, the variable importance and respective partial dependency plots for the most important variables in each model were extracted and visualized. In RF regression, the most common way to extract the variable importance is by the increase in Mean Squarer Error (iMSE). To compute the iMSE for a given feature, its values are randomly permuted and the internal RF performance metric, the Out of Bag (OOB) error is computed. Important variables are expected to significantly decrease model performance if permuted, reporting high values of iMSE [65, 71, 72]. For prediction, we used a 100-m grid resolution with variables aggregated at that level. Even though higher resolutions have been used such as 10 meters [6], 100 ms was a reasonable scale for mapping intra-urban PfPR_2_10_, capturing neighborhood variability and also matching with the spatial data used (i.e., population and land-use at the street block level).

To gain a deeper understanding of the urban malaria prevalence, we adjusted our predicted estimates according to the underlying population based on the population distribution maps. In each of the cities, we multiplied the predicted PfPR_2_10_ with the population of each grid cell to obtain the predicted number of infected people. Afterwards, we summarized this information at the administrative level that the population model was trained at. Finally, we computed population adjusted PfPR_2_10_ estimates, by dividing the total predicted number of infected people aggregated of a census unit with its total population as in previous works [73–77]. The census delineation for Dar es Salaam was an aggregated version of the administrative level 5 of the 2002 census, and was computed through k-means cluster analysis [55]. This was done to find suitable training formations for the population distribution models resolution and as such, these units do not represent official administrative levels. For Kampala, we made use of the 2002 level 4 census delineation.

### Hardware

The data processing and model training was performed with two Intel^®^ Xeon^®^ CPU E5-2690 (2 processors of 2.90 GHz, 16 cores and 32 processing threads) having 96 GB of RAM.

## Results

### Variable selection and importance

**Fig. 4:**
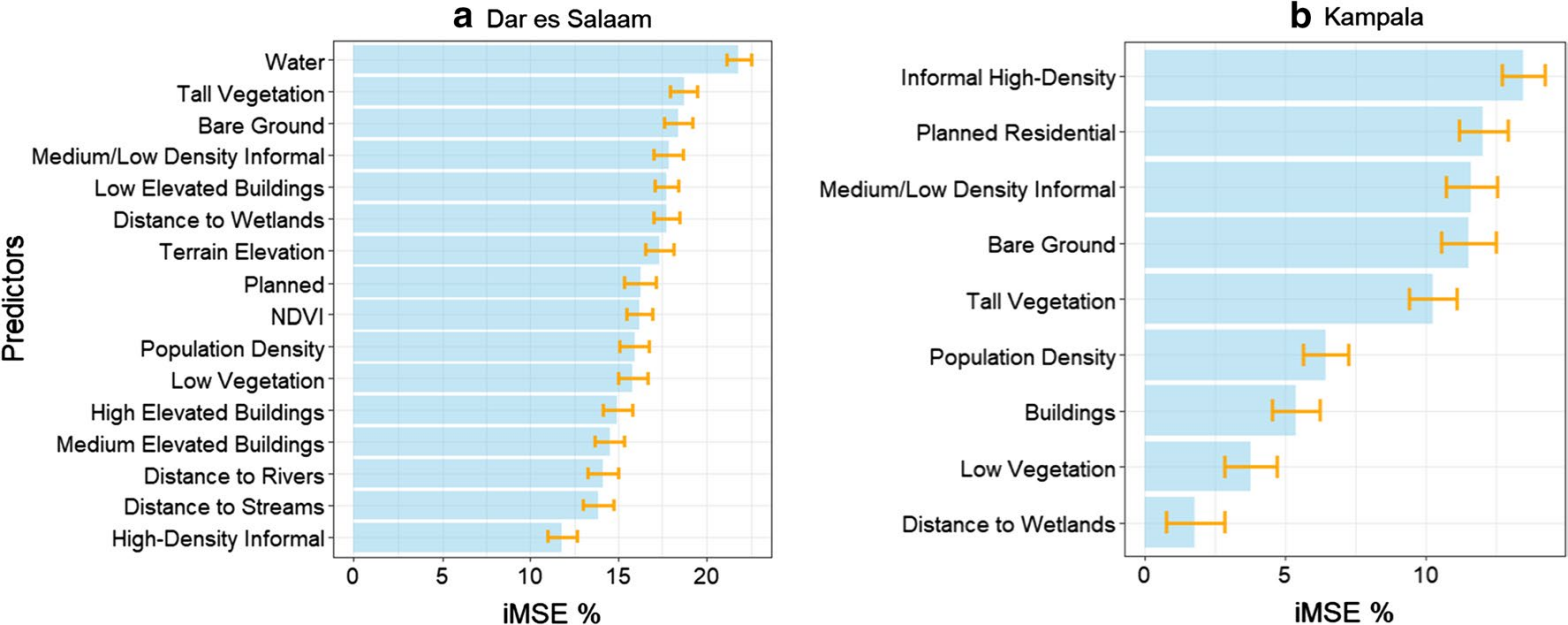
Variable importance across 100 model runs using all data points. **a** Dar es Salaam and **b** Kampala

Using the results of the VSURF as an anchor, we filtered out variables that had minimal or zero influence for the task of predicting PfPR_2_10_. Notably, in the case of Dar Es Salaam all initial features were kept, while in Kampala three features were dropped (SRTM, NDVI and the proportion of “water” class coming from the LC map). This could be explained both by the fact that the lower thematic detail of the Kampala LC information coupled with minimal coverage of inland water in the imagery. Figure 4 presents the variables used and their importance derived from the RF regressor for Dar es Salaam and Kampala. To account for uncertainty, we extracted the average importance over one hundred model runs, along with the corresponding standard deviation using all data points. The proportion of water was the most important variable in the prediction of PfPR_2_10_ in Dar es Salaam, along with the proportion of tall vegetation, bare ground, distance to wetlands, medium density informal settlements and low elevated buildings. Four out of the six most important variables were derived from the LC map which indicates the importance of mapping the physical characteristics of the surface. On the other hand, in Kampala, the LU classes were dominant in terms of feature importance. The typology of street blocks (residential, informal) was the most discriminating predictor of PfPR_2_10_, followed by the land cover classes of bare ground and tall vegetation. Other variables such as the population density, building density, the proportion of low vegetation and the distance to wetlands, while still important, contributed to lower degrees.

**Fig. 5:**
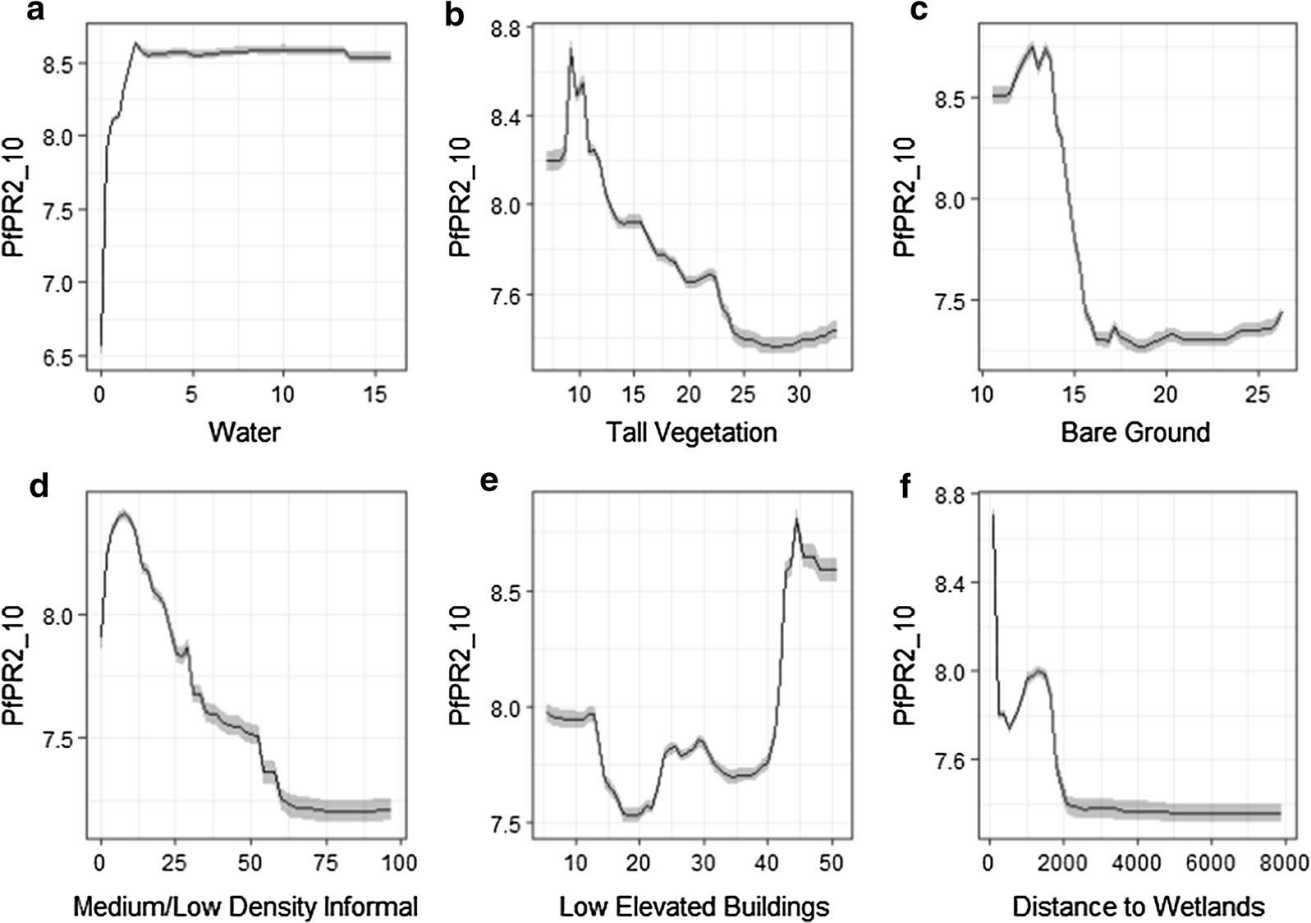
Partial dependency plots for the six most important model predictors in Dar es Salaam. The shaded area represents the standard deviation over 100 simulations using all data points. The x-axis in **a** to **e** represents proportions while in panel *f*, the x-axis units represent meters. The y-axis refers to the *Plasmodium falciparum* parasite rate standardized in the 2–10 years age range

To further illustrate these outcomes, we present the partial dependency plots averaged over 100 model runs for the six most important variables in each city. The dependency plots illustrate the response of PfPR_2_10_ with an increase in each explanatory variable, after adjusting for the effects of all other predictors. In Dar es Salaam (Fig. 5), the proportion of water was positively associated with PfPR_2_10_. After the threshold of roughly 2% of water, the response of malaria prevalence spiked in a positive manner and then levels off. This could be an indicator of small patches of inland water such as ponds, or riparian vegetation that is particularly humid, wetlands, or urban agriculture irrigation systems. Tall vegetation and bare ground are negatively associated. Moreover, there as a strong relationship between the distance from wetlands and a reduction in malaria prevalence. Low elevated buildings had a non-linear impact in PfPR_2_10_, where a negative trend is exhibited up to 40% and then the relationship became positive. Finally, medium/low density informal settlements demonstrated a negative relationship with malaria prevalence. This can be explained as this residential class represents the average and most common building type in Dar es Salaam.

**Fig. 6:**
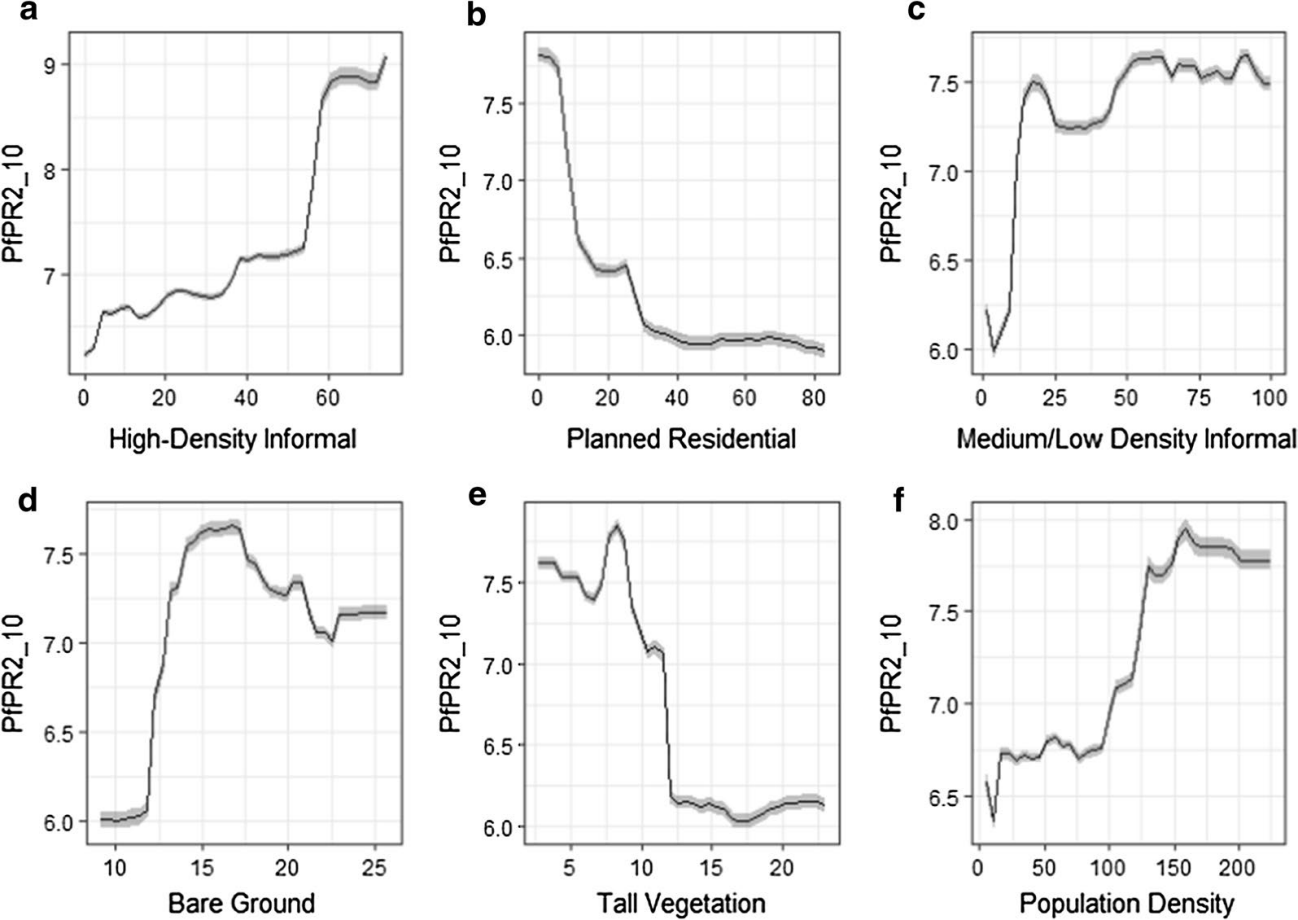
Partial dependency plots for the six most important model predictors in Kampala. The shaded area represents the standard deviation over 100 simulations using all data points. The x-axis in **a** to **e** represents proportions while in panel *f*, the x-axis units represent population per hectare. The y-axis refers to the *Plasmodium falciparum* parasite rate standardized in the 2–10 years age range

In Kampala, high-density informal settlements reported a strong positive relationship with malaria prevalence (Fig. 6). Different to Dar es Salaam, the proportion of medium/low informal settlements and bare ground exhibited a positive relationship with PfPR_2_10_ in Kampala. Meanwhile, planned residential blocks revealed a negative association, highlighting the importance of residential typology for identifying malaria hotspots. As in Dar Es Salaam, tall vegetation showcased a negative association with PfPR_2_10_.

### Model performance

The model performance metrics for the two cities are presented in Tables 2 and 3. Both models performed satisfactorily, with a median R^2^ of 0.39 and 0.43 in Kampala and Dar es Salaam, respectively. The model in Kampala indicated more dispersion across the bootstrap, and thus, increased uncertainty (Interquartile range of R^2^ = 0.45) while a smaller dispersion was noted for the Dar es Salaam model (Interquartile range of R^2^ = 0.33). With respect to the RMSE, Kampala exhibited a median RMSE of 5.45 while Dar Es Salaam a median score of 6.02. The MAE distribution for both cities was less dispersed than the RMSE, as it is not influenced as much by large error deviations. The median MAE values were 4.54 and 4.81 for Kampala and Dar es Salaam, respectively. The RMSE and MAE values should not be compared across cities as they are dependent on the amount of surveys and distribution of *Pf*PR_2–10_ values in each city.

**Table 2:** Descriptive statistics of the root mean squared error (RMSE), mean absolute error (MAE) and coefficient of determination (R^2^) model performance metrics for Kampala

Kampala			
	RMSE	MAE	R^2^
1st quartile	4.64	3.91	0.12
Median	5.45	4.54	0.39
Mean	5.53	4.59	0.38
3rd quartile	6.59	5.19	0.57

The RMSE and MAE refer to *Pf*PR_2–10_ values

**Table 3:** Descriptive statistics of the root mean squared error (RMSE), mean absolute error (MAE) and coefficient of determination (R^2^) model performance metrics for Dar es Salaam

Dar es Salaam			
	RMSE	MAE	R^2^
1st quartile	5.16	4.28	0.23
Median	6.02	4.81	0.43
Mean	6.05	4.88	0.39
3rd quartile	6.85	5.55	0.56

The RMSE and MAE refer to *Pf*PR_2–10_ values

### *PfPR*_*2_10*_ predictions

#### Dar es Salaam

**Fig. 7:**
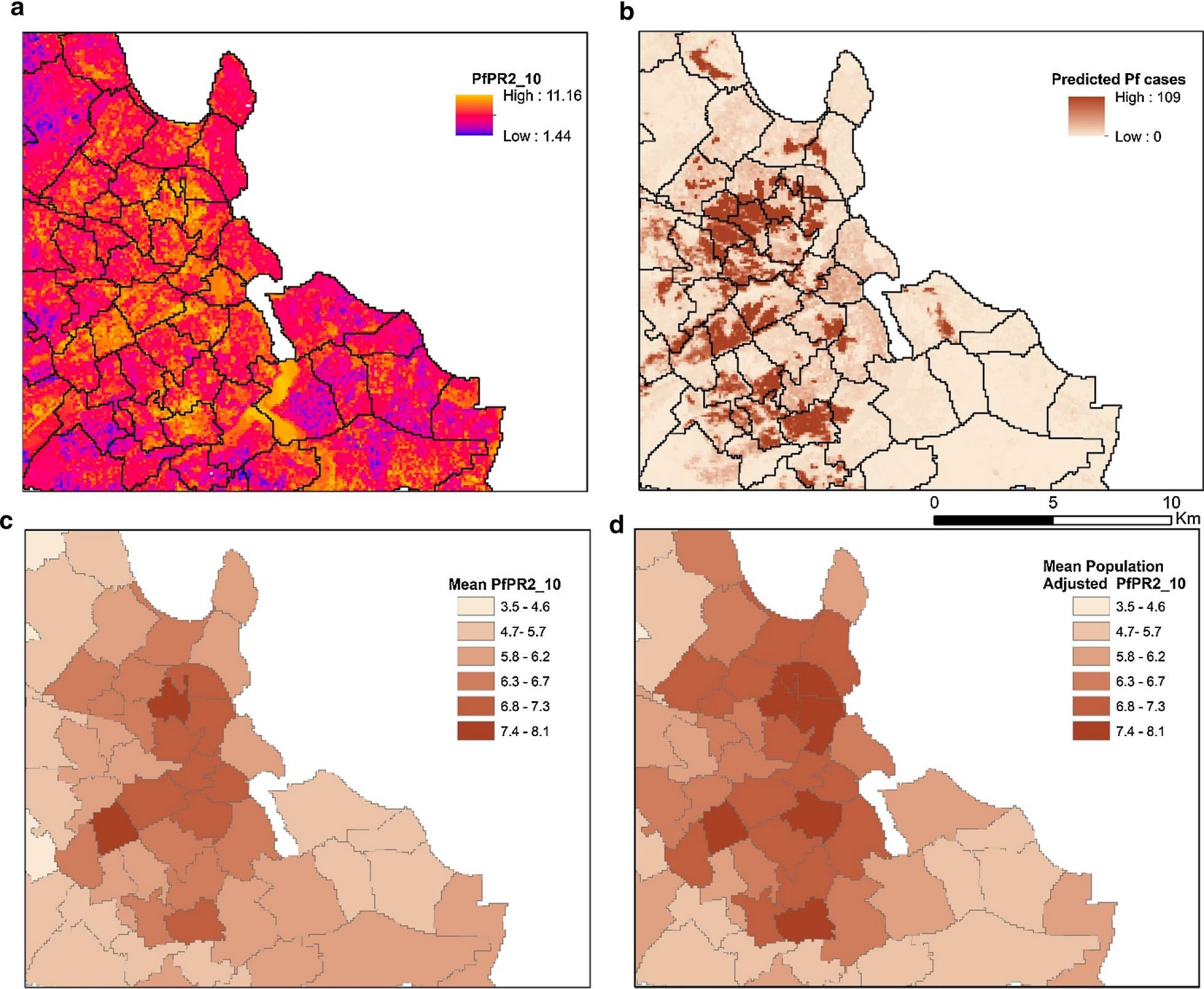
Model derivatives at a raster (**a**, **b**) and administrative (**c**, **d**) resolution for Dar es Salaam. **a** Predicted PfPR_2_10_ at a 100 m resolution, **b** number of predicted positive malaria cases at 100 m resolution using the distributed population grid, **c** Mean predicted PfPR_2_10_ at an aggregated version of the level 5 of the administrative level of the 2002 Tanzania Census and **d** Mean Population Adjusted PfPR_2_10_ at an aggregated version of the level 5 administrative level of the 2002 Tanzania Census

**Fig. 8:**
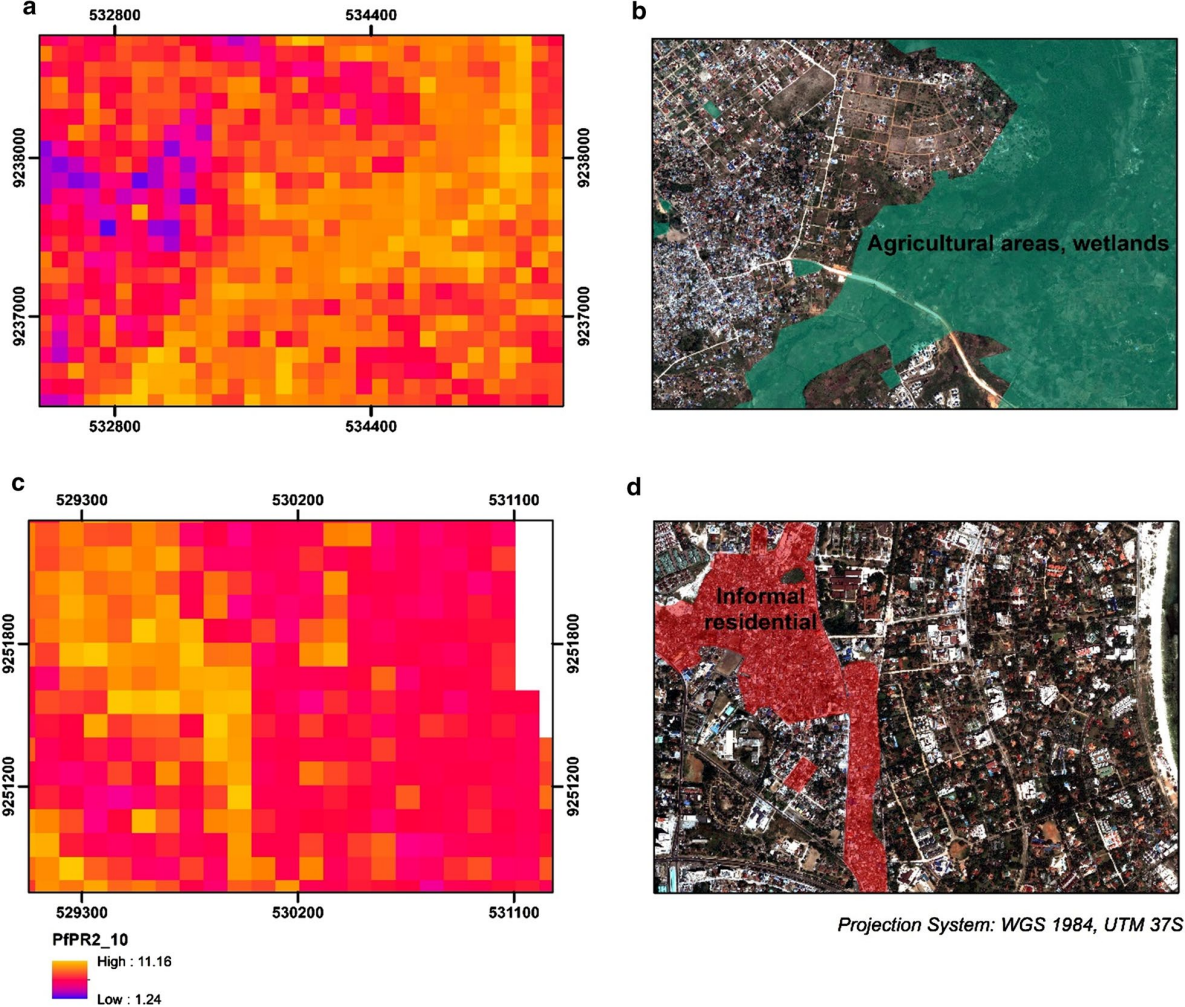
Predicted PfPR_2_10_ in 2 locations in Dar es Salaam. **a** Intensified urban agriculture across the Mbezi river, **c** distinction of estimates across the dense slums and planned neighborhoods. The second column (**b** and **d**), display the corresponding true color composite of the Pleiades satellite imagery. In **b** land-use classes of wetlands and agricultural are overlaid with shaded green. In **d** land-use blocks classified as informal settlements are overlaid with shaded red

As exhibited in Fig. 7, the predicted distribution of malaria prevalence in Dar es Salaam was diverse and did not follow a gradually increasing malaria risk as a function of the distance from the urban center. The spatial clustering of high *Pf*PR_2–10_ values appeared to be associated with the underlying physical and socioeconomic environment and develops across riparian vegetation, urban agriculture and highly dense informal settlements. When adjusted for population, the aggregated census polygons that contain highly dense informal settlements displayed high PfPR_2_10_ values, even if they were in the urban center. As Fig. 8 demonstrates, the predicted *PfPR*_*2_10*_ values in Dar es Salaam were lower across the wealthier planned neighborhoods of the urban center, while were significantly higher for the dense informal settlements (Fig. 8c), and regions of urban agriculture and wetlands (Fig. 8a).

#### Kampala

**Fig. 9:**
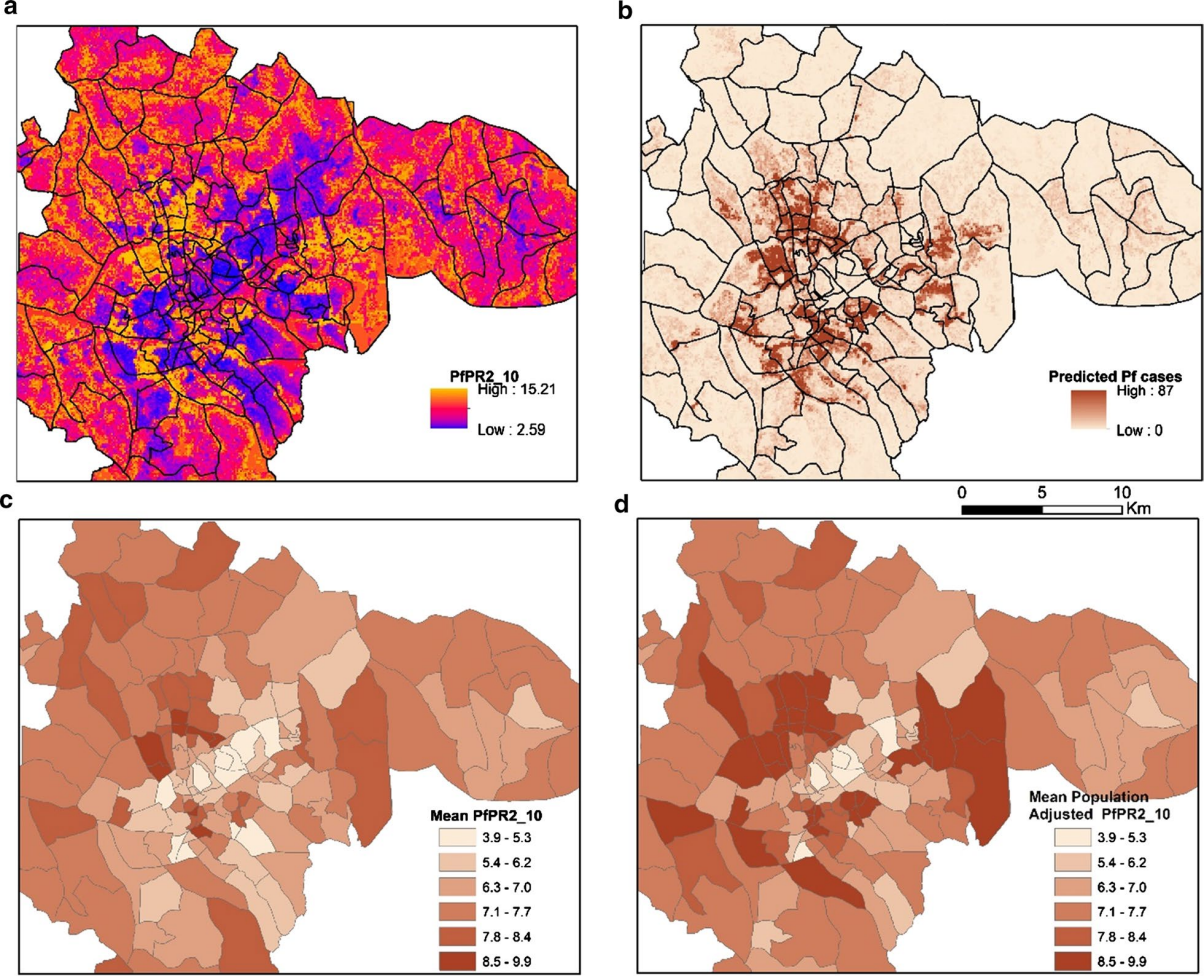
Model derivatives at a raster (**a**, **b**) and administrative (**c**, **d**) resolution for Kampala. **a** Predicted PfPR_2_10_ at a 100 meter resolution, **b** number of predicted positive malaria cases at 100 m resolution using the distributed population grid, **c** Mean predicted PfPR_2_10_ at the level 4 administrative level (2002 Uganda Census) and **d** Mean Population Adjusted PfPR_2_10_ at the level 4 administrative level (2002 Uganda Census)

**Fig. 10:**
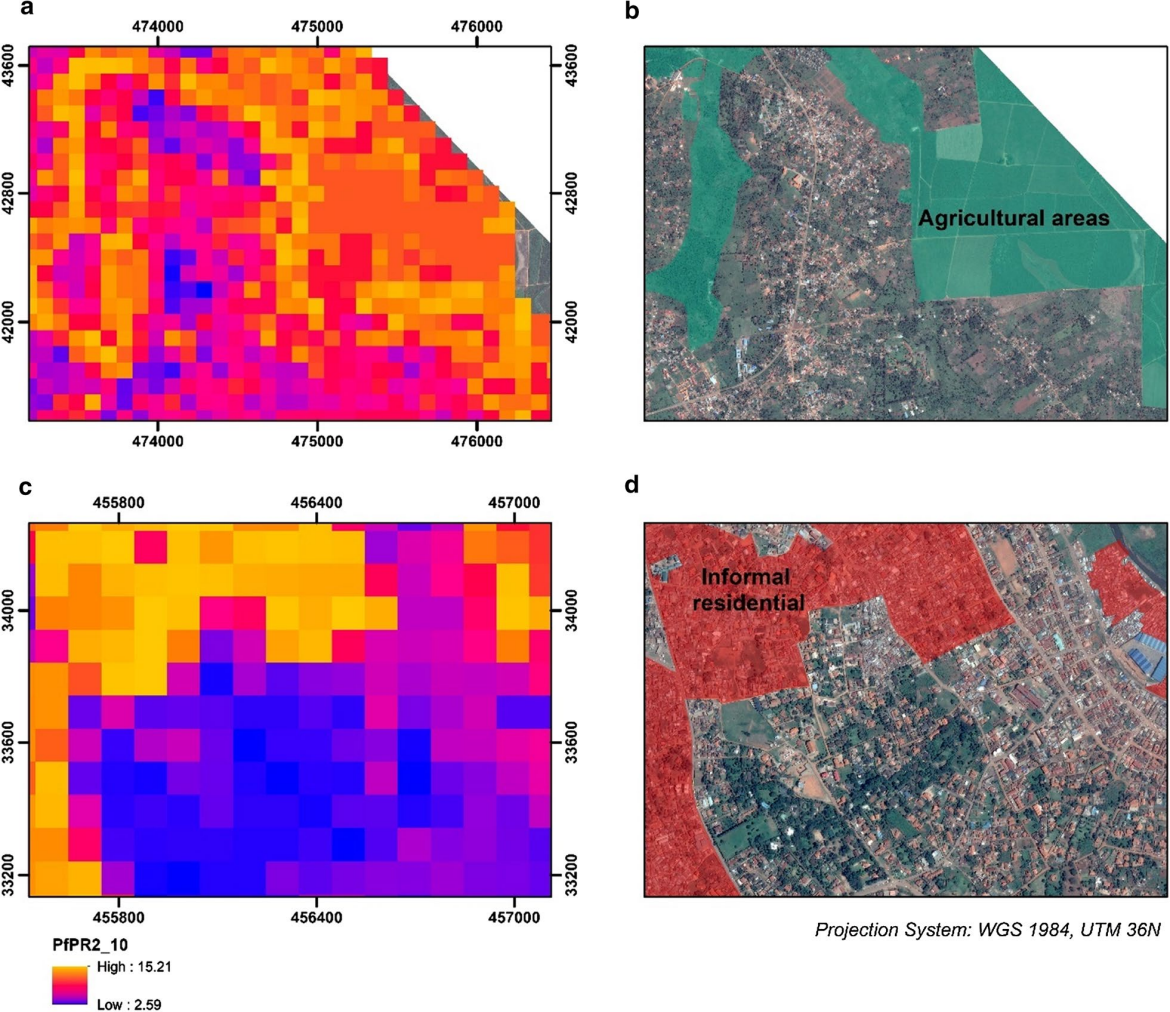
Predicted PfPR_2_10_ in 2 locations in Kampala. **a** Urban agriculture and **c** planned and informal residential neighborhoods. The second column (**b**, **d**), display the corresponding true color composite of the Pleiades satellite imagery. In **b** land-use classes of wetlands and agriculture are overlaid with shaded green. In **d** land-use blocks classified as informal settlements are overlaid with shaded red

In Kampala, the overall range of the parasite rate was higher than that of Dar es Salaam but with a different urban distribution. The highest values of predicted PfPR_2–10_ are in regions combining a set of physical and socio-economic criteria such as highly dense slums bordering wetlands. The overall predicted PfPR_2_10_ ranged from 2.6 to 15.2 at the grid level, while it decreased when summarized at an administrative level (3.9–9.9). Figure 9 illustrates these outputs across the main urban extent of Kampala. The population adjusted estimates signify increased risk across administrative units that contain large extents of highly populated slums, developed across large bodies of water, wetlands and humid vegetation. The risk in the planned and commercial center was significantly lower than in peri-urban regions whether accounting for population or not. Figure 10 shows snapshots of the predicted PfPR_2_10_ across different locations in Kampala. The model predicted increased values of PfPR_2_10_ in informal settlements (Fig. 10c), regions of urban agriculture, wetlands and swamps (Fig. 10a) while the risk was decreased in the planned residential areas.

### Spatial uncertainty

**Fig. 11:**
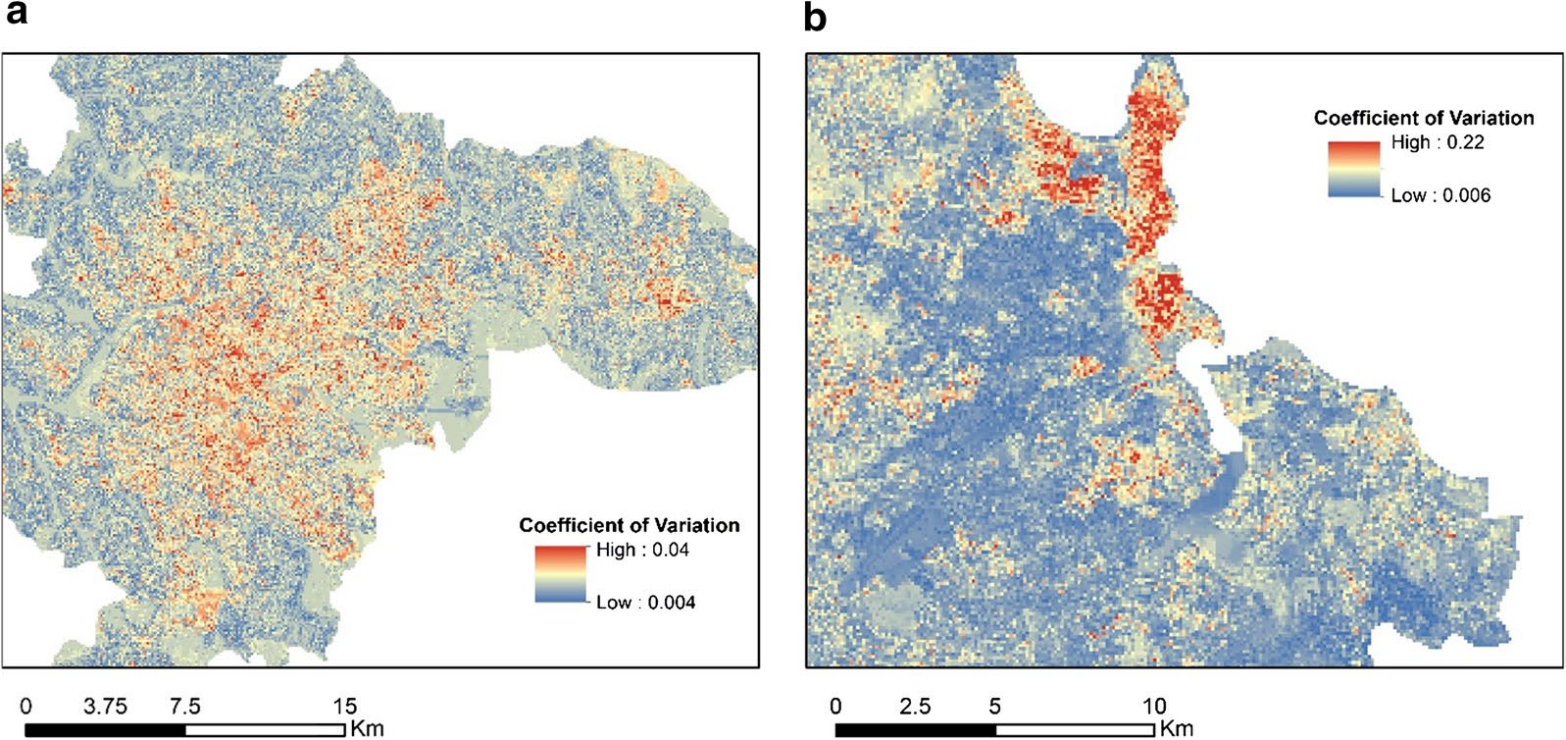
Coefficient of variation in **a** Kampala and **b** Dar Es Salaam. The coefficient of variation is computed on the predictions from 100 model runs in each city

Figure 11 presents the spatial distribution of the coefficient of variation (CV), computed on the predictions of the 100 model runs in each city. The CV values were low in both cities, indicating low spatial prediction uncertainty. Nonetheless, in relative terms across the spatial domain, some differences emerge. In Kampala, the CV was higher in the urban center with decreased values across the peri-urban regions, while in Dar es Salaam higher values of CV were clustered mostly at the planned residential neighborhoods.

## Discussion

### Relationship between satellite indicators and malaria prevalence

Previous research using remotely sensed datasets was able to distinguish malaria risk across the urban fabric in SSA cities, albeit using coarser resolution information [6, 9]. Given the nature of datasets and differences in objectives, only basic distinctions could be made (land cover classes such as building density and vegetation). Building density was in general negatively associated with malaria prevalence while vegetation exhibited positive associations. Although very informative, these models often neglect the importance of the underlying socio-economic relationship of different urban settlements with malaria risk. Here, our results support the notion that malaria prevalence is combination of physical factors such as urban land cover that favors the emergence of vector breeding sites, but also the type of surrounding communities (informal, formal), which provides an indication on their capability to cope with the burden of malaria. This aligns with previous work suggesting that malaria prevalence can be significantly higher in informal settlements in comparison to other urban landscapes [18, 19]. Additionally, we show that malaria prevalence can be linked to neighborhood location, where settlements located close to wetlands or agricultural fields were more affected. Recent evidence of increased insecticide resistance of malaria vectors in SSA cities is also fortifying the link between persistent malaria prevalence and urban agriculture [78]. The strength of VHR remotely sensed products resides in their ability to discriminate, with relative ease various types of urban communities based on their built-up characteristics (orientation, size, density, elevation). Moreover, with the latest advents in computer vision, analysis of very large areas (city extent) can be feasible with standard computers and open source software [49, 54]. Nonetheless, VHR imagery can be particularly costly for institutions in the Global South to acquire. Soon, we expect more VHR satellite data to be publicly distributed, as is the case already in some areas.

### Malaria data limitations and model assessment

The PfPR_2_10_ RF models are temporal composites of surveys ranging almost a decade, and consequently the temporal dimension was assumed stationary. We assumed that the extracted signal is mostly invariant as we focused on urban regions that have not undergone major transitional changes but might have expanded (i.e., large informal settlements, the planned residential center). Moreover, it was a necessary sacrifice in order to assemble a dataset large enough to capture the fine-scale spatial variability. Nonetheless, improvements in the modeling process can be expected if temporal effects are to be integrated. Furthermore, significant variations exist within the malaria datasets used in this study, with respect to sample sizes, survey locations and years of survey, which are likely to bias the results. Although, we attempted to mitigate these effects through stratified sampling and intense bootstrapping, a rigorous sensitivity analysis should be investigated when facing situations of multi-survey information as input to spatial models. Information regarding potential anti-malarial interventions was not incorporated as the number of surveys and information in both cities was limited. In future work, indicators pertaining to intervention campaigns should be investigated as some are already available at the national or regional levels [79]. As informative as they are, the model results should be used with caution and as complimentary material with other malaria sources and expert knowledge.

Our models explained about half of the variance, which is in range with predictive studies of malaria prevalence across various spatiotemporal scales [6, 80–84]. The results are expected to improve when information regarding human decisions and behavior is integrated, such as the use of insecticidal bed nets and type of infection (imported or acquired locally from rural–urban migration). Furthermore, we must acknowledge that the predictors used in this study cannot be considered absent of error. As with any LC and LU classification, there is a certain degree of misclassification error which can propagate in any subsequent analysis. This can be investigated further, using the maps of the coefficient of variation in the predictions. Areas that highlight hotspots of variation might indicate that a local refinement in the predictive variables of the LC and LU maps is needed and the classification process subsequently revisited. Alternatively, it might indicate uncertainties pertaining to the influence of the variables in the task of predicting PfPR_2_10_. Nonetheless, all the LC and LU products were produced with recent, state-of-the-art analysis and high degrees of accuracy, and validated by the high level of model performance and minimal spatial uncertainty. It should be noted that the models developed here are applicable only in an urban context and lose generalization ability in the rural or dominantly rural peri-urban regions.

### The importance of urban geography when addressing urban malaria

With respect to the predicted *PfPR*_*2_10*_ distributions in Dar es Salaam and Kampala we conclude there is not a straightforward urban–rural trend in malaria prevalence. As mentioned by previous urban malaria reviews [85], the underlying physical and socio-economic geography may dictate part of the malaria distribution in the city. This would explain why some cities exhibit hotspots of malaria prevalence in densely urbanized areas or intermediate zones rather than in surrounding, more rural regions [86, 87]. Aligning with these findings, our analysis demonstrated that central hotspots can be found when certain criteria are met, e.g., proximity to water bodies and humid, low, marsh-like vegetation, slums and agriculture. In Dar es Salaam, while low-elevated building density was mostly negatively associated with prevalence, a spike in PfPR_2–10_ was observed once its density exceeded a certain threshold-which can be described as a highly dense, informal settlement. On the contrary, in Kampala, the land-use predictors were the most dominant in terms of importance for predicting malaria prevalence as a clear dichotomy between slums and planned neighborhoods was exhibited, which was not obvious in Dar es Salaam. These variations could be explained by two main factors. First, the land cover product of Kampala is generally less detailed than that of Dar es Salaam since it contains only a single building density class. Given that stereoscopic images were used for the LC classification in Dar es Salaam, the building elevation was extracted, offering more discrimination capabilities. Second, there exists intrinsic historical differences with respect to the way each city has been built and developed. Kampala exhibits a clearer pattern of clustered, wealthier areas built in elevated topography, with slums developed around them. In Dar es Salaam, most of the city can be considered to have a more informal nature, and as such, the discriminatory power of the LU map might be more limited—at least to the level that was used in this study. Notably, the data extracted from OSM in Dar es Salaam were highly predictive and should be considered as an additional source of information when they have enough degree of completeness for a given study area.

### Intra-urban human population distribution maps as an additional tool to address urban malaria

To our knowledge this was the first study making use of fine-scale population data distributed through VHR information in order to adjust the PfPR_2_10_ estimates across two cities. As population density varies greatly across the urban fabric, efforts should be made to not only present the malaria prevalence as an abstract variable, but according to the underlying population at risk. It should also be emphasized that the population information used in both cities comes from a census carried out in 2002 and a temporal mismatch between the imagery and population counts can exist, even though relative patterns are expected to be similar. Nonetheless, population projection techniques can be applied to simulate both population and malaria cases in future dates.

### Future prospects

This work also serves as a call for the intensification of geolocated urban malaria surveys and their dissemination, while not neglecting privacy issues. With almost half of the SSA population predicted to be residing in cities by 2030 [88], understanding and mapping malaria prevalence across the various urban environments is of utmost importance for building more resilient cities. Our study suggests that more attention should be paid to informal settlements. The second point of note relates to secondary urban areas (SUA’s) in SSA. Most of the malaria research is focused on rural communities or main urban centers of economic growth. Nonetheless, SUA’s currently absorb about 75% of rural–urban migration and their growth rates can be considerably higher compared to the already large urban centers [89]. Zimmer et al. [90], analyzed SUA’s in 8 southern African countries and concluded that they account for about half the urban population. These secondary cities are undergoing severe transformations. However, not much is known about them either from an epidemiological or a geographical perspective, with a large part of available information coming through studies employing satellite remote sensing [90]. In order to reduce and eliminate urban malaria in the coming years and to encourage sustainable urbanization, these cities should become a focus of interest for the research and policy-making community to prevent situations that might lead to high degrees of sustained and persistent intra-urban malaria prevalence. Along with the development of a systematic ground survey network in these forthcoming urban centers, the use of remote sensing should be heavily exploited. Due to the high transferability of malaria models based on earth observation datasets even with a very limited number of ground data available, hotspots of malaria prevalence could be detected, facilitating evidence-based allocation of resources and enhancing evidence-based policy making in these cities.

## Conclusions

This research provides a framework to predict intra-urban malaria at a fine scale spatial resolution, coupling machine learning algorithms, very-high-resolution satellite derived indicators, and geospatial and survey data. Focusing on Dar es Salaam and Kampala as case studies, we conclude that the predictive dataset appears to be robust for modelling the intra-urban spatial distribution of malaria prevalence across large scales. Within both cities, urban malaria prevalence is not evenly distributed and varies intrinsically across the urban fabric. Informal settlements, urban agriculture and locations near wetlands and riparian vegetation are highlighted as potential hotspots. Population adjusted estimates indicate higher prevalence values in highly populated administrative units. Finally, the outcome of this work further encourages the use of satellite data to understand and investigate urban malaria enhancing evidence-based policy making and control efforts in SSA cities.

## Supplementary information

**Additional file 1** Methodological information regarding the creation of the land-cover, land-use and population products that were used as input to the malaria models.

**Additional file 2** Descriptive Statistics of parasite prevalence surveys assembled in Dar es Salaam and Kampala, respectively.

## Data Availability

The datasets that support the conclusions of this manuscript are deposited in the open access Zenodo scientific repository (https://zenodo.org/record/3871497#.XtTWPPZuKDA). The raw satellite imagery is property of AIRBUS DS, France, all rights reserved. The OpenStreetMap data used in this are publicly accessible and copyrighted under the mention “©OpenStreetMap contributors, CC BY-SA”. The Shuttle Radar Topography Mission information were extracted from https://www.usgs.gov/.

## Abbreviations

LU: Land use
LC: Land cover
NDVI: Normalized vegetation difference index
UMCP: Urban Malaria Control Program
PfPR_2_10_: Age standardized malaria parasite prevalence
OSM: OpenStreetMaps
CV: Coefficient of variation
RF: Random forest
VSURF: Variable selection using random forests
SSA: Sub-Saharan Africa
VHR: Very-high-resolution
