# Supplementary material for: Modelling and mapping the intra-urban spatial distribution of Plasmodium falciparum parasite rate using very-high-resolution satellite derived indicators
Source: Int J Health Geogr. 2020 Sep 21;19:38. doi: 10.1186/s12942-020-00232-2 (PMC7504835; doi:10.1186/s12942-020-00232-2)
Supplement: Supplementary file 1 — Additional file 1 Methodological information regarding the creation of the land-cover, land-use and population products that were used as input to the malaria models. [file 12942_2020_232_MOESM1_ESM.docx]

In this Additional file, we provide additional methodological information regarding the creation of the land-cover, land-use and population products that were used as input to the malaria models.

1. Land cover (LC) maps

The LC maps for Kampala and Dar es Salaam are derived from the semi-automated methodological framework of Grippa et al (1). It is an open source processing chain utilizing GRASS GIS (2), R and Python programming languages synthesized in a Jupyter Notebook format (Supplementary Figure 1). The documentation, metadata, and code of the processing chain can be found in Github (<https://github.com/tgrippa/Opensource_OBIA_processing_chain>). The main elements of the proposed framework are Geographic Object-Based Image Analysis, machine learning algorithms and LC training data created through Computer Assisted Photo Interpretation (CAPI). The overall accuracy (OA) of the LC products for Kampala and Dar es Salaam was 86% (7 classes) and 90% (9 classes), respectively, assessed through the out of bag error of a random forest classifier.

**Additional Figure S1**. Excerpt of the Jupyter notebook documenting the LC classification processing chain that was used in this study. Figure taken from Grippa et al.(1).

1. Land-use (LU) maps

The LU maps were created through a publicly available, semi-automated processing chain utilizing a PostGIS database system, OpenStreetMap (OSM) data and machine learning algorithms (3). The Python code for the street block processing can be found in the following GitHub repository (<https://github.com/ANAGEO/OSM_Streetblocks_extraction>). As visualized in Supplementary Figure 2, OSM linear elements are transformed into street blocks through vector processing in a PostGIS database. Afterwards, the street blocks are classified according to their land use through a supervised classification exercise using satellite information as proxies. The computation of the various spatial metrics based on satellite imagery can be found in the following GitHub repository (<https://github.com/tgrippa/Street_blocks_features_computation>). The overall accuracies for the LU products for Kampala and Dar es Salaam were 81% and 84%, respectively.


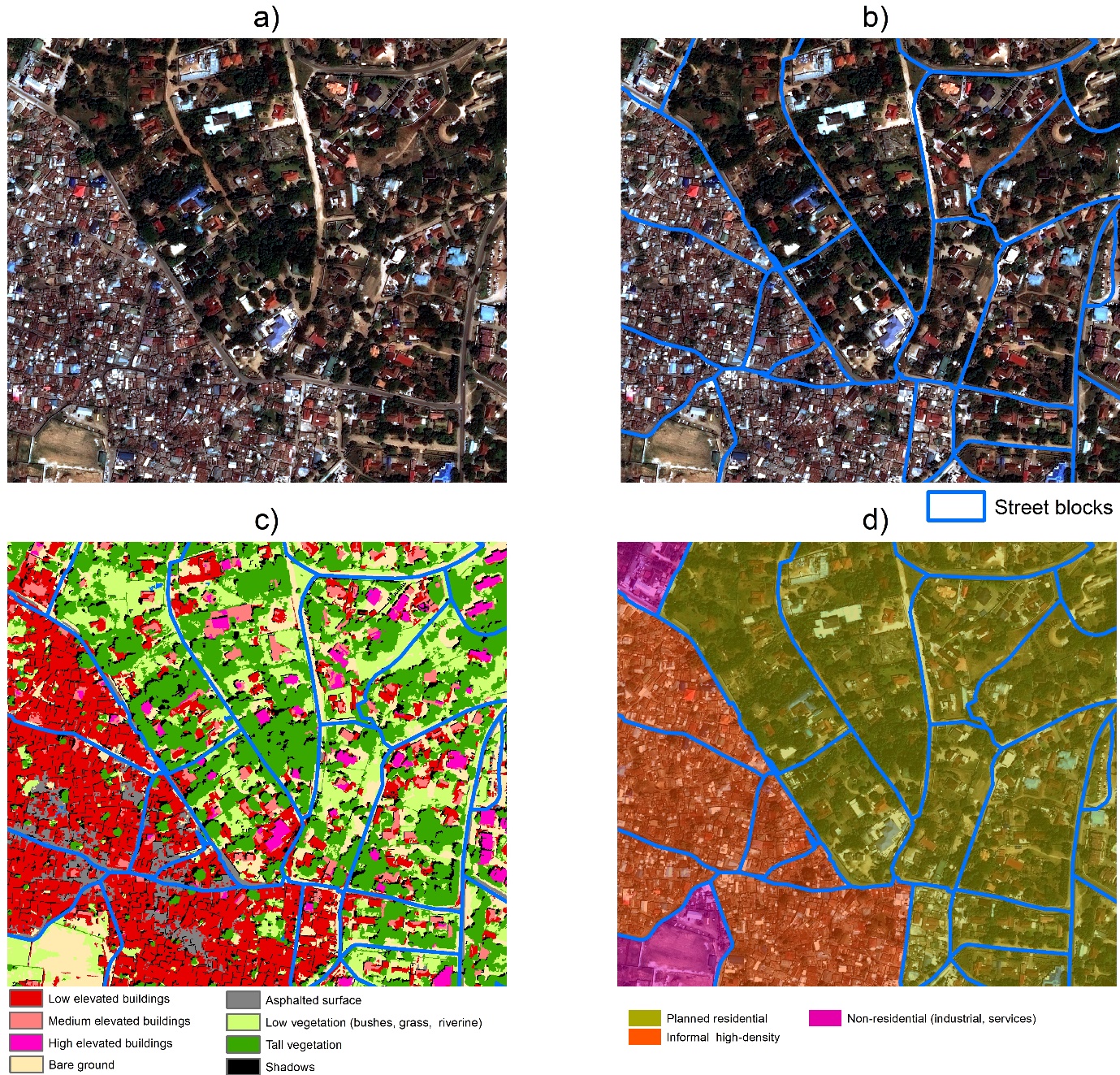


**Additional Figure S2**. Land-use mapping framework in Dar es Salaam, Tanzania. Land cover information is aggregated at the street block level and through a supervised machine learning classification, a land use label is assigned to each block.

1. Population maps

The population maps were produced through the open-access processing chain of Grippa et al. (4). The chain employs dasymetric population distribution methods tailored for very-high-resolution land cover and land use information. The code and documentation of the processing chain can be found in the following GitHub repository (<https://github.com/tgrippa/Dasymetric_mapping_using_GRASSGIS>). The coefficient of determination through independent validation for the population models of Kampala and Dar es Salaam was 0,76 and 0,63, respectively.

References

1. Grippa T, Lennert M, Beaumont B, Vanhuysse S, Stephenne N, Wolff E. An Open-Source Semi-Automated Processing Chain for Urban Object-Based Classification. Remote Sens [Internet]. 2017;9(4):358. Available from: http://www.mdpi.com/2072-4292/9/4/358

2. Neteler M, Bowman MH, Landa M, Metz M. GRASS GIS: A multi-purpose open source GIS. Environ Model Softw. 2012;31:124–30.

3. Grippa T, Georganos S, Zarougui S, Bognounou P, Diboulo E, Forget Y, et al. Mapping Urban Land Use at Street Block Level Using OpenStreetMap, Remote Sensing Data, and Spatial Metrics. ISPRS Int J Geo-Information. 2018;7(7).

4. Grippa T, Linard C, Lennert M, Georganos S, Mboga N, Vanhuysse S, et al. Improving Urban Population Distribution Models with Very-High Resolution Satellite Information. Data [Internet]. 2019;4(1). Available from: http://www.mdpi.com/2306-5729/4/1/13
