# Supplementary material for: Modelling and mapping the intra-urban spatial distribution of Plasmodium falciparum parasite rate using very-high-resolution satellite derived indicators
Source: Int J Health Geogr. 2020 Sep 21;19:38. doi: 10.1186/s12942-020-00232-2 (PMC7504835; doi:10.1186/s12942-020-00232-2)
Supplement: Supplementary file 2 — Additional file 2 Descriptive Statistics of parasite prevalence surveys assembled in Dar es Salaam and Kampala, respectively. [file 12942_2020_232_MOESM2_ESM.docx]

**Additional Table S1.** Descriptive Statistics of parasite prevalence surveys assembled in Dar es Salaam.

| **District/Ward** | **Number of Cluster** | **Total Individuals examined** | **Start Year** | **Start Year** | **Age Range** | **PfPR_2-10_ Range** | **Mean *Pf*PR_2-10_** |
| --- | --- | --- | --- | --- | --- | --- | --- |
| **Temeke District** | **25** | **5180** | **2005** | **2005** | **0 - 16** | **0 - 23,34** | **7,90** |
| *Kigamboni* | 1 | 102 | 2014 | 2014 | 4 - 16 | - | 0 |
| *Charambe* | 1 | 102 | 2014 | 2014 | 4 - 16 | - | 1,03 |
| *Miburani* | 3 | 891 | 2005 | 2005 | 0 - 5 | 2,4 - 18,71 | 10,43 |
| *Mtoni* | 4 | 1041 | 2005 | 2005 | 0 - 5 | 1,03 - 18,6 | 9,7 |
| *Kurasini* | 1 | 564 | 2007 | 2007 | 0 - 5 | - | 17,07 |
| *Azimio* | 3 | 816 | 2005 | 2005 | 0 - 5 | 12,3 - 23,34 | 18,84 |
| *Sandali* | 1 | 132 | 2014 | 2014 | 4 - 16 | - | 0 |
| *Vijibweni* | 1 | 93 | 2014 | 2014 | 4 - 16 | 1,13 | 1,13 |
| *Chang'ombe* | 1 | 102 | 2014 | 2014 | 4 - 16 | - | 2,06 |
| *Mbagala Kuu* | 1 | 103 | 2014 | 2014 | 4 - 16 | - | 1,02 |
| *Mji Mwema* | 2 | 66 | 2006 | 2006 | 0 - 4,9 | 4,48 - 14,01 | 9,24 |
| *Chamazi* | 1 | 43 | 2014 | 2014 | 4 - 16 | - | 0 |
| *Kijichi* | 1 | 102 | 2014 | 2014 | 4 - 16 | - | 2,06 |
| *Keko* | 3 | 891 | 2005 | 2005 | 0 - 5 | 6,39 - 11,58 | 8,87 |
| *Kilakala* | 1 | 132 | 2014 | 2014 | 4 - 16 | - | 1,59 |
| **Kinondoni District** | **29** | **5004** | **2005** | **2014** | **0 - 16** | **0 - 18,35** | **6,77** |
| *Magomeni* | 4 | 983 | 2005 | 2014 | 0 - 16 | 4,68 - 18,35 | 13,39 |
| *Mabibo* | 1 | 23 | 2008 | 2008 | 0,4 - 4,3 | - | 4,68 |
| *Goba* | 1 | 94 | 2014 | 2014 | 4 - 16 | - | 2,23 |
| *Kawe* | 2 | 113 | 2008 | 2014 | 0,1 - 16 | 0 - 3,21 | 1,60 |
| *Makuburi* | 1 | 17 | 2008 | 2008 | 0,5 - 4,9 | - | 0 |
| *Kimara* | 2 | 114 | 2008 | 2014 | 0,2 - 16 | - | 0 |
| *Mikocheni* | 3 | 611 | 2005 | 2014 | 0 - 16 | 0 - 15,62 | 9,24 |
| *Mbezi* | 1 | 97 | 2014 | 2014 | 4 - 16 | - | 1,08 |
| *Hananasif* | 1 | 98 | 2014 | 2014 | 4 - 16 | - | 0 |
| *Ndugumbi* | 4 | 926 | 2005 | 2008 | 0 - 16 | 0 - 17,53 | 7,69 |
| *Tandale* | 1 | 98 | 2014 | 2014 | 4 - 16 | - | 0 |
| *Mwananyamala* | 3 | 805 | 2005 | 2007 | 0 - 16 | 13,36 - 16,12 | 14,58 |
| *Msasani* | 1 | 99 | 2014 | 2014 | 4 - 16 | - | 2,12 |
| Mzimuni | 3 | 902 | 2005 | 2007 | 0 - 5 | 4,9 - 12,82 | 9,12 |
| *Wazo* | 1 | 24 | 2008 | 2008 | 0,2 - 4,5 | - | 0 |
| **Ilala District** | **36** | **5598** | **2005** | **2005** | **0 - 16** | **0 - 24,66** | **8,45** |
| *Ukonga* | 3 | 129 | 2006 | 2006 | 0 - 4,9 | 0 - 3,86 | 2,03 |
| *Buguruni* | 5 | 1051 | 2005 | 2005 | 0 - 16 | 0 - 21,75 | 7,58 |
| *Upanga Mashariki* | 1 | 105 | 2014 | 2014 | 4 - 16 | - | 0 |
| *Kiwalani* | 1 | 113 | 2014 | 2014 | 4 - 16 | - | 0 |
| *Segerea* | 1 | 106 | 2014 | 2014 | 4 - 16 | - | 0 |
| *Kitunda* | 1 | 106 | 2014 | 2014 | 4 - 16 | - | 1,98 |
| *llala* | 7 | 860 | 2005 | 2005 | 0 - 5 | 0 - 24,66 | 12,12 |
| *Mchikichini* | 4 | 898 | 2005 | 2005 | 0 - 5 | 2,24 - 11,1 | 9,79 |
| *Vingunguti* | 8 | 1205 | 2005 | 2005 | 0 - 16 | 1,98 - 22,89 | 9,38 |
| *Kipawa* | 3 | 819 | 2005 | 2005 | 0 - 5 | 16,29 - 22,18 | 19,49 |
| *Kivule* | 1 | 100 | 2014 | 2014 | 4 - 16 | - | 0 |
| *Gongolamboto* | 1 | 106 | 2014 | 2014 | 4 - 16 | - | 0,99 |
| **Total** | **90** | **15782** | **2005** | **2016** | **0 - 16** | **0 – 24,66** | **7,76** |

**Additional Table S2.** Descriptive Statistics of parasite prevalence surveys assembled in Kampala.

| **Division/**District | **Number of Cluster** | **Total Individuals examined** | **Start Year** | **End Year** | **Age Range** | **PfPR_2-10_ Range** | **Mean *Pf*PR_2-10_** |
| --- | --- | --- | --- | --- | --- | --- | --- |
| **Central Division** | **6** | **674** | **2007** | **2014** | **6 - 16** | **0 - 7,96** | **3,79** |
| *Kisenyi II* | 1 | 108 | 2014 | 2014 | 6 - 16 | - | 1,97 |
| *Kisenyi III* | 1 | 107 | 2014 | 2014 | 6 - 16 | - | 7,96 |
| *Kololo I* | 1 | 107 | 2014 | 2014 | 6 - 16 | - | 7,96 |
| *Kololo III* | 1 | 100 | 2014 | 2014 | 6 - 16 | - | 1,06 |
| *Mengo* | 1 | 91 | 2014 | 2014 | 6 - 16 | - | 0 |
| **Kawempe** | **6** | **837** | **2005** | **2014** | **1 - 16** | **2,15 - 20,96** | **8,62** |
| *Bwaise I* | 1 | 60 | 2006 | 2006 | 5 - 15 | - | 20,96 |
| *Kyebando* | 1 | 98 | 2014 | 2014 | 6 - 16 | - | 2,17 |
| *Makerere I* | 1 | 99 | 2014 | 2014 | 6 - 16 | - | 2,15 |
| *Mbererwe* | 1 | 102 | 2014 | 2014 | 6 - 16 | - | 6,26 |
| *Mulago III* | 2 | 478 | 2005 | 2005 | 1 - 10 | 8,35 - 14,75 | 11,55 |
| **Kira** | **3** | **116** | **2010** | **2012** | **0 - 4,9** | **1,19 - 16,01** | **8,6** |
| *Kira* | 2 | 88 | 2010 | 2012 | 0 - 4,9 | 0 - 2,38 | 1,19 |
| *Kirinya* | 1 | 28 | 2010 | 2010 | 0 - 4,9 | 0 | 16,01 |
| **Makindye** | **4** | **403** | **2014** | **2014** | **6 - 16** | **1,13 - 6,08** | **3,63** |
| *Ggaba* | 1 | 102 | 2014 | 2014 | 6 - 16 | - | 5,22 |
| *Katwe II* | 1 | 102 | 2014 |  | 6 - 16 | - | 2,09 |
| *Kisugu* | 1 | 105 | 2014 | 2014 | 6 - 16 | - | 6,08 |
| *Luwafu* | 1 | 94 | 2014 | 2014 | 6 - 16 | - | 1,13 |
| **Nabweru** | **2** | **69** | **2010** | **2010** | **0 - 4,9** | **9,61 - 13,18** | **11,39** |
| *Kazo Nabweru* | 1 | 35 | 2010 | 2010 | 0 - 4,9 | - | 9,61 |
| *Nansana* | 1 | 34 | 2010 | 2010 | 0 - 4,9 | - | 13,18 |
| **Nakawa** | **5** | **535** | **2014** | **2014** | **6 -16** | **0 - 5,6** | **3,14** |
| *Banda* | 1 | 125 | 2014 | 2014 | 6 -16 | - | 0 |
| *Bukoto Li* | 1 | 121 | 2014 | 2014 | 6 -16 | - | 3,52 |
| *Kyanya* | 1 | 94 | 2014 | 2014 | 6 -16 | - | 3,4 |
| *Luzira* | 1 | 100 | 2014 | 2014 | 6 -16 | - | 3,19 |
| *Mbuya I* | 1 | 95 | 2014 | 2014 | 6 -16 | - | 5,6 |
| **Nangabo** | **2** | **83** | **2012** | **2012** | **0 - 4,9** | **6,23 - 21,46** | **13,84** |
| *Wampeewo* | 2 | 83 | 2012 | 2012 | 0 - 4,9 | 6,23 - 21,46 | 27,69 |
| **Nsangi** | **2** | **61** | **2010** | **2010** | **0 - 4,9** | **4,15 - 9,89** | **7,02** |
| *Kasenge* | 1 | 27 | 2010 | 2010 | 0 - 4,9 | - | 4,15 |
| *Kyengera* | 1 | 34 | 2010 | 2010 | 0 - 4,9 | - | 9,89 |
| **Rubaga Division** | **5** | **433** | **2006** | **2014** | **5 - 16** | **0,87 - 24,45** | **10,03** |
| *Kabowa* | 1 | 97 | 2014 | 2014 | 6 - 16 | - | 4,39 |
| *Kasubi* | 1 | 60 | 2006 | 2006 | 5 - 15 | - | 24,45 |
| *Namirembe* | 1 | 122 | 2014 | 2014 | 6 -16 | - | 0,87 |
| *Nateete* | 1 | 100 | 2014 | 2014 | 6 -16 | - | 1,06 |
| *Rubaga* | 1 | 54 | 2006 | 2006 | 5 - 15 | - | 19,4 |
| **Ssabagabo-Makindye** | **3** | **90** | **2010** | **2010** | **0 - 4.9** | **0 - 13,18** | **5,83** |
| *Busabala* | 1 | 34 | 2010 | 2010 | 0 - 4.9 | - | 13,18 |
| Masajja | 1 | 26 | 2010 | 2010 | 0 - 4.9 | - | 4,31 |
| Ndejje | 1 | 30 | 2010 | 2010 | 0 - 4.9 | - | 0 |
| **Wakiso** | **2** | **75** | **2010** | **2012** | **0 - 4,9** | **2,8 - 6,9** | **4,85** |
| *Buloba* | 1 | 35 | 2012 | 2012 | 0 - 4,9 | - | 6,4 |
| *Nakabugo* | 1 | 40 | 2010 | 2010 | 0 - 4.9 | - | 2,8 |
| **Total** | **39** | **3215** | **2005** | **2014** | **0 - 16** | **0 - 24,45** | **6,76** |
